# Canine Gouging: A Taboo Resurfacing in Migrant Urban Population

**DOI:** 10.1155/2015/727286

**Published:** 2015-07-21

**Authors:** Anila Virani Noman, Ferranti Wong, Ravikiran Ramakrishna Pawar

**Affiliations:** ^1^Centre for Oral Growth & Development, Paediatric Dentistry, Queen Mary University of London, Barts and The London School of Medicine and Dentistry, Institute of Dentistry, Turner Street, London E1 2AD, UK; ^2^Dental and Maxillofacial Radiology, Queen Mary University of London, Barts and The London School of Medicine and Dentistry, Institute of Dentistry, Turner Street, London E1 2AD, UK

## Abstract

Cosmopolitan cities have become a pool of migrants from different parts of the world, who carry their cultural beliefs and superstitions with them around the globe. Canine gouging is a kind of infant oral mutilation (IOM) which is widely practiced among rural population of Africa where the primary tooth bud of the deciduous canine is enucleated. The belief is that the life threatening illnesses in children like vomiting, diarrhoea, and fevers are caused by worms which infest on tooth buds. This case report is of a 15-year-old Somalian born boy, who presented at the dental institute with intermittent pain in his lower right permanent canine which was associated with a discharging intra oral buccal sinus. The tooth was endodontically treated and then restored with composite. General dental practitioners need to be vigilant when encountered with tooth presenting unusual morphology, unilateral missing tooth, and shift in the midline due to early loss of deciduous/permanent canines. Identification of any such dental mutilation practice will need further counselling of the individual and family members. It is the duty of every dental professional to educate and safeguard the oral and dental health of general public.

## 1. Introduction

Cosmopolitan cities have become a pool of migrants. With the influx of people from different corners of the world, cultural beliefs and superstitions travel with them around the globe. This paper emphasises on a taboo of tooth enucleation practiced in the rural populations of sub-Saharan and eastern Africa. Such practice has been published in the literature previously but it is important to reiterate that these misbeliefs are still practiced in the urban population. It is not uncommon to encounter such cases in a cosmopolitan city like London.

Canine gouging is a kind of infant oral mutilation (IOM) which is widely practiced among rural population of Africa where the primary tooth bud of the deciduous canine is enucleated. The belief is that the life threatening illnesses in children like vomiting, diarrhoea, and fevers are caused by worms which infest tooth buds. Some believe that the tooth follicle itself resembles worms as they are soft, unmineralized mass of tissue. These teeth have been known by different names as Ebinyo, or Ebino, nylon or vinyl teeth, killer teeth, and Lugbara [1–6]. The other reasons for such practice have been general malaise or ill health, itching gums, crying with an unknown cause, failure to suckle, and sometimes even being performed as a preventive measure to keep illness at bay [7, 8]. Enucleation of the tooth bud is believed to cure the infants from any such ailment. This practice has run in families of local “healers” who practice this without using any anaesthesia or antiseptics. Another belief as reported by Kenyan Massai women believe that bovine calf is not prone to diarrhoea or febrile illnesses as it does not possess canines and hence removing the canine can cure diseases [6, 9, 10].

The technique involves rubbing of herbs or ashes got from burring leaves on the gums to prevent the child from getting disease. This process is called silencing [6]. If the child is ill, then the process is usually conducted by middle aged Massai women (in Kenya), older women, family member, priest, teachers, religious healers, or the tribal head [7, 11, 12]. The instrument used to enucleate the tooth can be finger nails, pointed knife, hot needle, bicycle spokes, rusty nails, or wires [7, 8, 12]. Although there are reported cases of deaths of young children following canine gouging due to septicaemia, anaemia, meningitis, osteomyelitis, and tetanus, the practice still continues [13].

The origin of this practice is unknown but could be speculated on the fact that incising the gingiva with a lancet to help an erupting tooth would relieve pain and discomfort to the patient. In 1575 Ambriose Pare, a French army surgeon, incorporated this method to help the problem of “breeding tooth.” In 1668, Francois Mauriceau insisted to use lancet instead of knife or coins to conduct the procedure. In 1742, Joseph Hurlock encouraged such practice to prevent child deaths caused because of teething [7, 14]. It would be ironical to understand if such a practice transformed to gouging.

One of the earliest literature reports on canine gouging comments on such practice in the pagan tribes of Nilotic Sudan in 1932. The Shilluk tribes practiced a custom of removing the deciduous lower incisors. The Acholi tribe from Uganda were known to remove the lower canine tooth buds. It is speculated that such a practice was initiated and spread in Uganda by Lugbara tribe. Pindborg in 1969 was the first who shed light on the topic and its relation to the superstitious beliefs in Uganda [2, 6]. This is not the first time the western world has come across such practice in the African continent.

Evidence of canine gouging practice has been documented from different parts of Africa like Uganda, Angola, Tanzania, Somalia, Kenya, Sudan, and Nigeria and has been in the literature [1, 3, 4, 9, 13, 15–22]. In Cape Flats (Western Cape) in South Africa a culture of removing incisors has been followed for the last 60 years. The incidence of such practice was higher in lower income areas [23]. The prevalence of this practice has been different from place to place ranging between 15 and 80% in children below the age of 4 years [12]. This practice has also been reported in non-African countries like Maldives, United States, New Zealand, Israel, and Sweden especially in migrant population [12, 24–27]. There are also reports of such practice in the United Kingdom by Somalian and Ugandan migrants who are currently residents of UK [1].

The aim of this paper is to present a complication of the practice of primary canine extraction in young children and spread awareness among the general dental practitioners to be vigilant of such practice and plan the treatment accordingly. It is also important to counsel the individual and their family to prevent performing such practice. It is the duty of every dental professional to educate and safeguard the oral and dental health of the general public.

## 2. Case Report

This is a case report of a 15-year-old Somalian born boy, who presented at the paediatric dentistry department. Consent was obtained from the patient and parent to use the following information for this publication. Patient had intermittent pain in his lower right permanent canine which was associated with a discharging intraoral buccal sinus. He was concerned with the aesthetics of this tooth. On further questioning it was revealed that at the age of five the boy suffered from high temperature, diarrhea, and vomiting. The local dentist in Somalia performed a traditional tooth enucleation procedure to cure the boy from illness. His family history revealed he has seven younger siblings. No such enucleation was seen performed on other siblings as all others were born in the UK.

**Figure 1:**
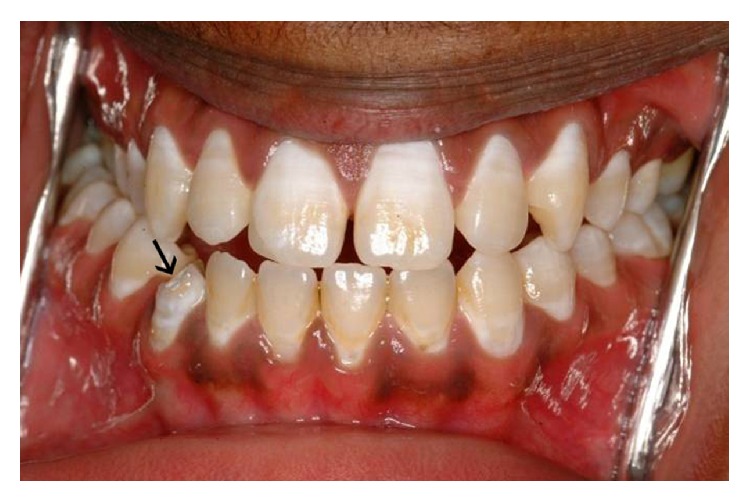
Before treatment: intra-oral views showing hypoplastic LR3.

**Figure 2:**
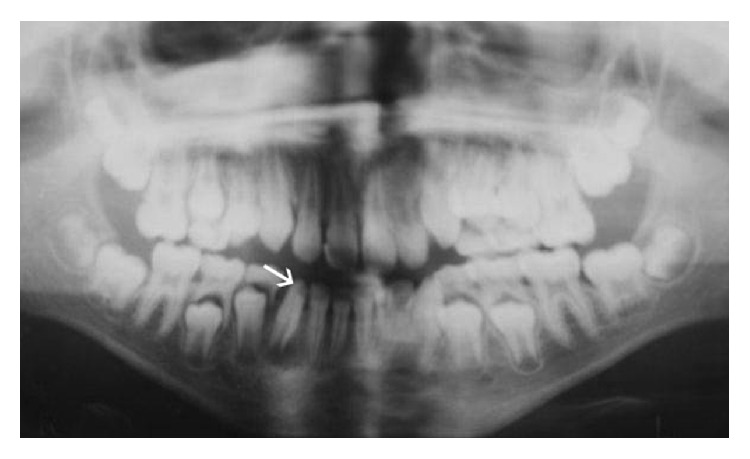
Orthopantomogram taken at initial presentation showing periapical changes in the LR3.

**Figure 3:**
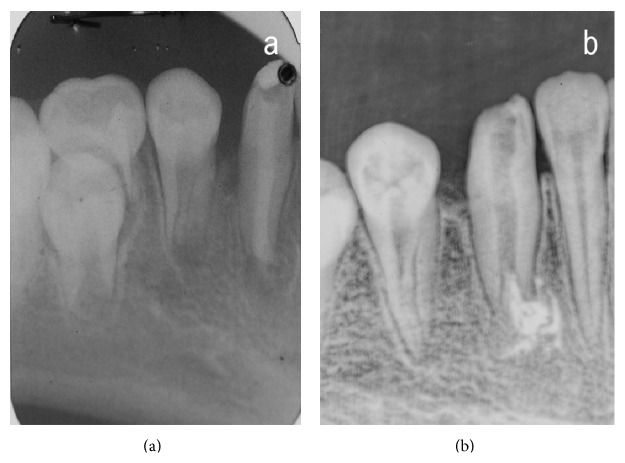
Lower periapical radiograph. (a) Monitoring phase (2007). (b) Treatment phase (May 2009).

The patient was medically fit and well. On clinical examination the lower right permanent canine appeared hypoplastic as shown in the pretreatment figure (Figure 1). Draining sinus was noted in the buccal sulcus. The tooth presented grade 1 mobility. In addition to this all the permanent teeth showed fluorosis. A panoramic and periapical radiograph taken showed coronal tooth loss and demonstrated an open apex (Figures 2 and 3(a)). Patient was given an option to undergo endodontic treatment or extraction. Endodontic treatment of this tooth was the most suggestive treatment and was carried out in successive appointments (Figure 3(b)). This was followed by fibre post/everstick post and coronal structure was restored by indirect composite buildup (belleglass composite).

**Figure 4:**
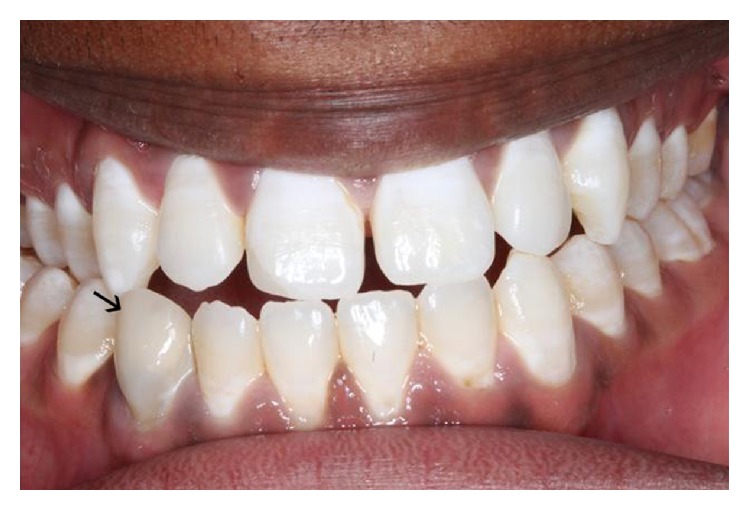
After treatment: following composite buildup and bleaching.

The permanent teeth were finally bleached to improve his dental aesthetics as shown in the posttreatment figure (Figure 4). The patient and the parent were given additional counselling about the practice of canine gouging and its ill effects on the teeth.

## 3. Discussion

The present case shows the result of a previous enucleation procedure and how it has led to the changes in the permanent tooth. The practice of canine gouging may result in trauma or infection to the permanent canine tooth bud which can leave the tooth to be hypoplastic or completely atrophied. Hypoplastic teeth are known to be predisposed to caries [10, 28]. An early loss of deciduous tooth especially when it is unilateral can result in shift of the midline [29]. Rather sinister consequences are excessive bleeding, infections, osteomyelitis of jaws, noma, tetanus, meningitis, aspiration bronchopneumonia, HIV infections, hepatitis, and even death [8].

There are other dental mutilation practices including transformation of teeth by shaving of the teeth, placing jewellery on tooth, gold crowns on normal sound anterior teeth, tattooing on the lips, and uvulectomy. Canine gouging is one of such dental mutilation practices.

Some of these practices have been culturally determined [30]. A deeper cause for such superstitious beliefs is lack of education, poverty, lack of belief in medical practice, and failure of good medical infrastructure. With the high prevalence of infectious diseases like diarrhoea, tuberculosis, HIV infection, and malaria and inadequate medical supplies and reduced access to trained dentist, it is easy to access local “traditional healers” through traditional rituals. It is always seen that such practices are common in lower income group.

Matee and Helderman [18] studied the prevalence of nylon teeth practice in Tanzania in subjects (*n* = 3267) within the age group of 3 to 19 years. 95% of the missing teeth were canine [17]. Hiza and Kikwilu (1992) accounted such extraction in Tanzania to be 37.4% in children (*n* = 262) and found 99.4% teeth involved to be canines bilatarally [3]. In another study by Kikwilu and Hiza (1997) they examined children with missing primary teeth, scars, or wounds in gingivae and found such practice was more in villages where traditional healing with extraction of tooth buds was recently reported. Such prevalence was reported to be 60% in a group of children (*n* = 1052) [4]. Similar studies conducted by Hassanali et al. (1995) in Kenya showed the occurrence was as high as 87% in children (*n* = 95) between the age group of 6 months to 2 years. The peak age group which reported such practice has been 4–18 months of age [9]. A study conducted by Ngilisho LA et al. in 1994 in five villages of Tanzania found that most of the traditional villagers were trained by their father or grandfather and the tradition has passed on in family [7]. The traditional healers also believed that they treated on average at least 3 dental patients per month.

It is important to note that the absence of primary canine in these ethnic populations could indicate the practice of canine gouging. Such practice can be endemic and sometimes associated with other rituals practiced by the community. One needs to be aware of such practice and associated social factors and its effect on the dental and physical and psychological well-being of the patient. A close examination of the dentition, absence of deciduous or permanent canine, scarring along the area, and loss of alveolar ridge height could indicate previous practice of canine gouging [31].

In the present case we came across similar observation and a comprehensive history revealed the practice of canine enucleation in the past.

The risk of morbidity and mortality from these practices needs to be explained to the patients especially from these ethnic backgrounds. Educating and counselling pregnant women and parents of young children about canine gouging and associated risk it imposes on the health and life of the child need to be addressed. Its ill effect on the primary and secondary dentition needs to be explained. As a dental professional one needs to be cautious in identifying such unknown tradition and provide the right guidance and advice.

## 4. Conclusion

Canine gouging or canine enucleation is a superstitious belief and is still practice in some migrant populations. General dental practitioners need to be aware of such practice and observe patient with such problem when coming across unilateral missing tooth. A comprehensive history from the patient or parent can be helpful in identifying the root cause for such missing tooth. It is also important to counsel the individual and their family to prevent performing such practice.

## References

[1] Dewhurst S. N., Mason C. (2001). Traditional tooth bud gouging in a Ugandan family: a report involving three sisters. *International Journal of Paediatric Dentistry*.

[2] Pindborg J. J. (1969). Dental mutilation and associated abnormalities in Uganda. *The American Journal of Physical Anthropology*.

[3] Hiza J. F., Kikwilu E. N. (1992). Missing primary teeth due to tooth bud extraction in a remote village in Tanzania. *International Journal of Paediatric Dentistry*.

[4] Kikwilu E. N., Hiza J. F. R. (1997). Tooth bud extraction and rubbing of herbs by traditional healers in Tanzania: prevalence, and sociological and environmental factors influencing the practices. *International Journal of Paediatric Dentistry*.

[5] Batwala V., Mulogo E. M., Arubaku W. (2007). Oral health status of school children in Mbarara, Uganda. *African Health Sciences*.

[6] Gollings J., Longhurst R. (2011). *Infant Oral Mutilation*.

[7] Johnston N. L., Riordan P. J. (2005). Tooth follicle extirpation and uvulectomy. *Australian Dental Journal*.

[8] Woodruff A. W., El Suni A., Kaku M., Adamson E. A., Maughan T. S., Bundru N. (1983). Infants in Juba, Southern Sudan: the first six months of life. *The Lancet*.

[9] Hassanali J., Amwayi P., Muriithi A. (1995). Removal of deciduous canine tooth buds in Kenyan rural Maasai. *East African Medical Journal*.

[10] Caufield P. W., Li Y., Bromage T. G. (2012). Hypoplasia-associated severe early childhood caries—a proposed definition. *Journal of Dental Research*.

[11] Mutai J., Muniu E., Sawe J., Hassanali J., Kibet P., Wanzala P. (2010). Socio-cultural practices of deciduous canine tooth bud removal among Maasai children. *International Dental Journal*.

[12] Edwards P. C., Levering N., Wetzel E., Saini T. (2008). Extirpation of the primary canine tooth follicles: a form of infant oral mutilation. *The Journal of the American Dental Association*.

[13] A/Wahab M. M. (1987). Traditional practice as a cause of infant morbidity and mortality in Juba area (Sudan). *Annals of Tropical Paediatrics*.

[14] Ashley M. P. (2001). It's only teething...a report of the myths and modern approaches to teething. *British Dental Journal*.

[15] Halestrap D. J. (1971). Indigenous dental practice in Uganda. *British Dental Journal*.

[16] Bataringaya A., Ferguson M., Lalloo R. (2005). The impact of ebinyo, a form of dental mutilation, on the malocclusion status in Uganda. *Community Dental Health*.

[17] Jones A. (1992). Tooth mutilation in Angola. *British Dental Journal*.

[18] Matee M. I., van Palenstein Helderman W. H. (1991). Extraction of 'nylon' teeth and associated abnormalities in Tanzanian children. *African Dental Journal*.

[19] Ngilisho L. A., Mosha H. J., Poulsen S. (1994). The role of traditional healers in the treatment of toothache in Tanga Region, Tanzania. *Community Dental Health*.

[20] Rodd H. D., Davidson L. E. (2000). ‘Ilko dacowo:’ canine enucleation and dental sequelae in Somali children. *International Journal of Paediatric Dentistry*.

[21] Baba S. P., Kay E. J. (1989). The mythology of the killer deciduous canine tooth in southern Sudan. *The Journal of Pedodontics*.

[22] Aderinokun G. A., Onadeko M. O. (1990). Prematurely erupted deciduous teeth in a Nigerian baby—a case report. *African Dental Journal*.

[23] Friedling L. J., Morris A. G. (2005). The frequency of culturally derived dental modification practices on the Cape Flats in the Western Cape. *SADJ*.

[24] Fitton J. S. (1993). A tooth ablation custom occurring in the Maldives. *British Dental Journal*.

[25] Holan G., Mamber E. (1994). Extraction of primary canine tooth buds: prevalence and associated dental abnormalities in a group of Ethiopian Jewish children. *International Journal of Paediatric Dentistry*.

[26] Erlandsson A.-L., Bäckman B. (1999). A case of dental mutilation. *ASDC Journal of Dentistry for Children*.

[27] de Beavis F. O. V., Foster A. C., Fuge K. N., Whyman R. A. (2011). Infant oral mutilation: a New Zealand case series. *New Zealand Dental Journal*.

[28] Hong L., Levy S. M., Warren J. J., Broffitt B. (2009). Association between enamel hypoplasia and dental caries in primary second molars: a cohort study. *Caries Research*.

[29] Martins-Júnior P. A., Marques L. S. (2012). Clinical implications of early loss of a lower deciduous canine. *International Journal of Orthodontics*.

[30] Johnson C. L. (2015). *The Cultural Modification of Teeth*.

[31] Goracci G., Marci F., Negri P. L., Treccani A. (1983). Aspects of dental fluorosis in subjects from regions with water rich in fluorine and their classification. *Minerva Stomatologica*.

